# Adequacy of Informed Consent in Elective Surgical Procedures: A Study in a Navi Mumbai Tertiary Care Centre

**DOI:** 10.7759/cureus.41777

**Published:** 2023-07-12

**Authors:** Amit Patil, Shreyas Chawathey, Adel Malim

**Affiliations:** 1 Forensic Medicine and Toxicology, All India Institute of Medical Sciences, Patna, Patna, IND; 2 Anaesthesiology, Critical Care and Pain, Homi Bhabha National Institute, Tata Memorial Hospital, Mumbai, IND; 3 College of Medicine, Dr D Y Patil Medical College, Hospital, and Research Centre, Navi Mumbai, IND

**Keywords:** decision making., adequacy, medicolegal, ethics, autonomy, informed consent

## Abstract

Background

Informed consent (IC) is a voluntary authorisation given by a patient or research subject after fully comprehending the risks involved in various procedures and treatments. Though a patient may fulfill all the aspects of consent by completing an informed consent form (ICF), research indicates poor execution of the IC process by ill-informed patients with little comprehension. The present study was done on patients to assess their understanding and involvement in the consenting process, thereby providing insight into the adequacy and sufficiency of the IC process.

Materials and methodology

Patients undergoing elective surgical procedures were surveyed using a questionnaire to study whether the written informed consent (IC) process was adequately used in elective surgeries and to assess the patient’s understanding of the IC and whether the informed consent forms (ICF) used met the ethical and legal standards for this purpose. The questionnaire was administered to the patients by two surveyors. As per the inclusion/exclusion criteria, data was collected from 221 admitted patients who were planned to undergo or recently underwent various elective surgical/operative procedures. Descriptive analysis using frequency and percentages of the positive and negative responses was used to analyse the data.

Results

In 219 (99%) of the cases, informed consent was taken. Two hundred-eight patients (94.1%) understood the knowledgeable consent information, while 13 (5.9%) did not. Of the total 221 patients, more than 90% of patients were informed about the nature and indication of the surgery. The expected benefits were told to 83.25% of patients, while possible complications of the procedure were reported to 91 patients (41.2%). Of the total, 58.37 % of patients knew the type of anaesthesia used for elective surgery. Two hundred and sixteen (97.73%) patients favoured the informed consent process, and 213 (96.38%) were satisfied with the information provided in the consent form. The education status of the patient varied, with nearly 15.5% being illiterate while 35.3% being educated till high school. Patients undergoing surgical procedures must be explained the nature and indication of the proposed surgical treatment, including its benefits and risks. About 208 (94.1%) of the patients stated that they understood all the information provided in the ICF, and 213 patients (96.3%) were satisfied with it. Most patients (88.7%) exercised autonomy in deciding to undergo surgery. Ninety-seven percent of patients favoured the IC process, of which 38.46% believed informed consent has a medicolegal significance.

Conclusion

The present study revealed that a better understanding of the informed consent by the patients is a vital component of the process as it helps exercise autonomy in the decision-making process. However, the lack of information in the informed consent forms critically affects the quality and adequacy of the IC, thus posing ethical and legal challenges to genuinely informed consent.

## Introduction

Informed consent (IC) is a lawful and ethical doctrine derived from the principle of respect for autonomy. It is a legal term supported by jurisdiction and international laws. It is a voluntary authorisation given by a patient or research subject after fully comprehending the risks involved in various procedures and treatments [1]. IC requires full disclosure of all relevant information by the treating doctor that a competent patient will appreciate by understanding the facts, thereby offering a non-coerced and autonomous agreement to the proposed treatment [2].

Earlier studies have found that most IC procedures must be completed, and most patients need help recalling or understanding the content of the informed consent documents [3]. Though a patient may fulfill all the aspects of consent by completing an informed consent form (ICF) [4,5], research indicates poor execution of the IC process by ill-informed patients with little comprehension [6]. The cost of providing incomplete information about medical procedures may range from low patient satisfaction, poor adherence to the treatment, patient regret, and patient litigation against the doctors [7-14]. Reviews of the IC process of medical research and clinical trial participation also highlight serious shortcomings [15-17]. Evidence also suggests that patients have some general information or idea about the aim and nature of the procedure and specific risks thereof but are more interested in the outcome [18-22].

In general, valid informed consent must fulfill the following vital requirements [23-25]: (a) disclosure of adequate information; (b) capacity or competency to understand that information; (c) voluntary decision-making without coercion or force, or deception; (d) comprehension to understand the given information; (e) consent or agreement to the proposed treatment or procedure. 

The patient’s perspective concerning the above five key elements must be assessed to ensure genuinely informed consent. A systematic review has expressed concern about established and standardised measures of the consent process and has suggested severe limitations in the current approaches to the IC process both in measurement and clinical practice [26]. The present study was done on patients undergoing elective surgical procedures to assess their understanding and involvement in the consenting process, thereby providing insight into the adequacy and sufficiency of the IC process.

## Materials and methods

Study objectives and design

The primary objective was to study whether the IC process was adequately used in elective surgeries and to assess the patient’s understanding of the IC. The other purpose was to analyse the informed consent forms (ICF) content to see whether they met the ethical and legal standards. This cross-sectional observational study was done in an urban hospital over one year, from February 2016 to January 2017. Over the study period, as per the inclusion/exclusion criteria, data was collected from 221 admitted patients who were planned for undergoing or who recently underwent elective surgical/operative procedures in various surgical departments like General Surgery, Ophthalmology, Obstetrics and Gynaecology, Cardiothoracic, etc. The study was approved by the Institutional Ethics Committee of Dr D Y Patil Hospital, Navi Mumbai dated 10 September 2016 vide letter no. PDYMC/Ethics/Sept-04/2016.

Study procedure and setting

The study was conducted in a tertiary care teaching and research hospital. Patients undergoing elective surgical procedures were surveyed using a questionnaire to study whether the IC process was adequately used in elective surgeries and to assess the patient’s understanding of the IC. The questionnaire was administered to the patients by two surveyors who were medical students and co-investigators in this study. 

The questionnaire was adopted from a study done by Amir et al. [27] with certain modifications done according to the study’s objectives and the local population. A Marathi questionnaire was prepared as modified from the English version of the Amir et al. paper, in addition to the questionnaires available in Hindi and English.

The other goal was to analyse whether the informed consent forms (ICF) used met the ethical and legal standards for this purpose. To study this objective, the informed consent forms filled by the patients/participants were analysed for the presence/ absence of information, including whether the following details were mentioned: name of the patient, diagnosis, name of the procedure and related information, risks involved in the procedure, alternative procedures, complications arising out of the planned as well as alternative procedures as per a pre-validated checklist. As per the study objectives, the data capture sheet (Figure 2) framed for this study had three (03) parts: Part I contained socio-demographic details of the participants from their hospital records. Part II had questions regarding various components of informed consent. It had 15 questions to be responded to as either “Yes” or “No”. Finally, part III recorded information obtained from a checklist from Amir et al. study [27] about the completeness/adequacy of the ICF so that it conforms to ethical and legal standards.

The co-investigators interviewed and administered the questionnaire in English, Hindi, or Marathi to patients admitted to the hospital by surgeons of different specialities. Patients above the age of 18 years undergoing elective surgical procedures and willing to participate were included in the study. Non-consenting and minor-age patients were excluded from the study. Any procedure that is not an emergency and requires prior informed consent of the competent person (patient or their spouse or next of kin or a person legally authorised to provide informed consent on behalf of the patient) was considered to be an elective surgical procedure.

Data collection and analysis

Microsoft Excel and Statistical Package for Social Sciences (SPSS) version 22 (IBM Corp., Armonk, NY) were used to tabulate and analyse the data. Descriptive analysis using frequency and percentages of the positive and negative responses was used to analyse the data. In addition, the data regarding adequacy and completeness of information from the informed consent was collected as positive and negative responses and analysed using frequency and percentages. Tables and figures were used to present the findings.

## Results

**Figure 1:**
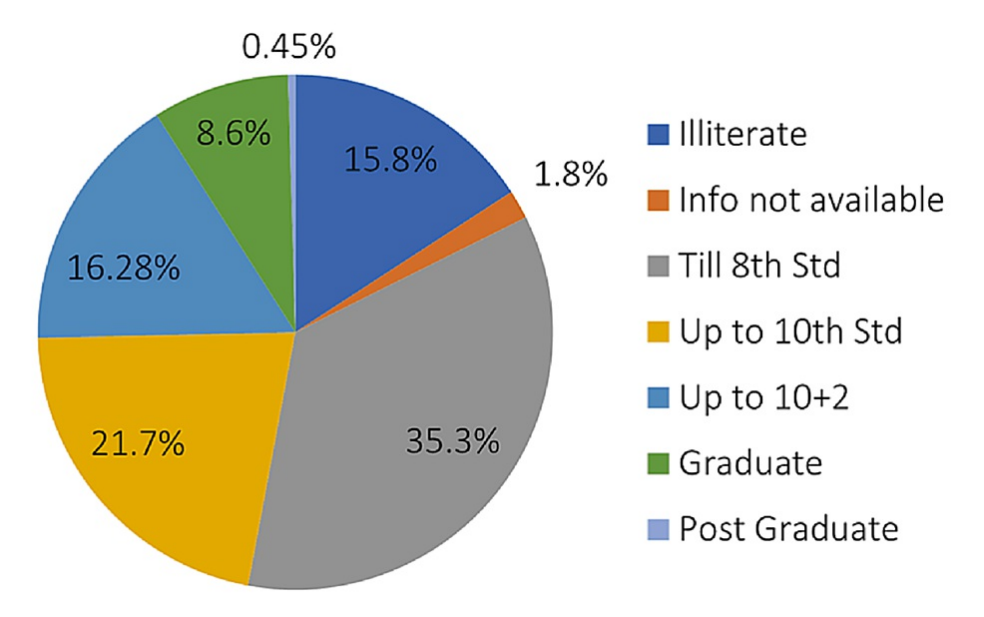
Education status of the participants

**Figure 2:**
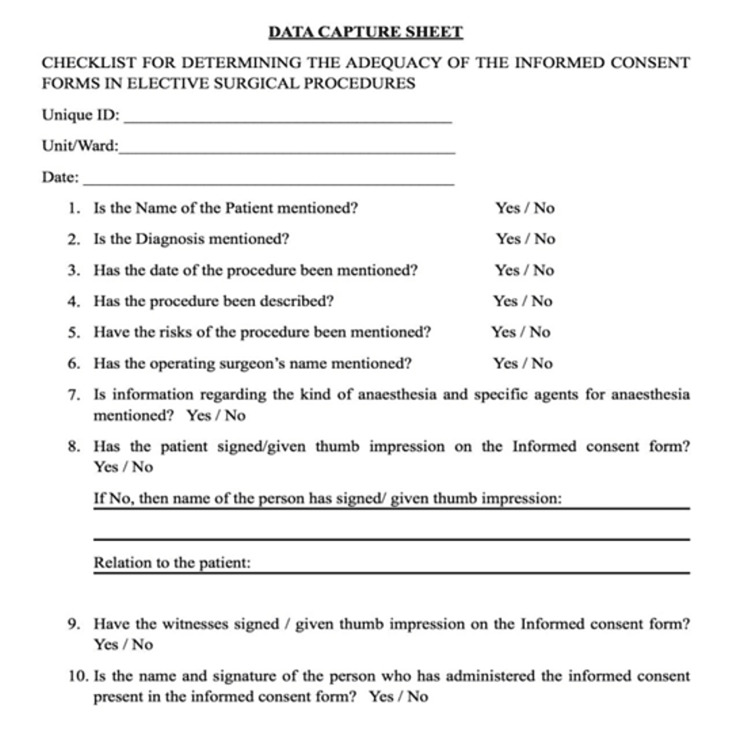
Data capture sheet

The patient population had the following characteristics: patients’ ages ranged from 18-80 years, with the average age being 41.72 years (Table 1). Female respondents were 134 (60.6%), and male respondents were 87 (39.4%), with male to female (M:F) ratio being 2:3. One hundred and twenty-six (57.1%) participants were from urban areas, and 95 (42.9%) were from rural areas. The participants; education details (Figure 1) revealed that 15.8% were illiterate, 21.7% studied till secondary school, 16.28% completed their higher secondary education, and only 9% of our patients were graduates and postgraduates.

**Table 1:** Participants' age in years

Age group (years)	No. of participants
18-27	59
28-37	40
38-47	32
48-57	49
58-65	24
More than 65	17
Total	221

The participant’s responses to the questionnaire are as given in Table 2. Informed consent for surgery was taken in 219 (99%) cases, while in two cases (1%), no informed consent forms were found on patient records. Of these, 208 patients (94.1%) understood the information in the informed consent form, while 13 (5.9%) did not. The information about the nature and indication of the surgery was given to 200 (90.5%) and 215 (97.28%) patients, respectively. The expected benefits and the possible complications of the elective procedure were informed to 184 (83.25%) and 91 (41.2%) patients. In case the surgery was not done, alternative treatments were informed to only 90 (40.72%) patients, while the possible complications if the procedure was not done were informed to 150 (67.87%) participants.

**Table 2:** Questionnaire responses by the participant

Questions	Yes	%	No	%
Was an informed consent taken?	219	99	2	1
Did you understand the information provided?	208	94.1	13	5.9
Were you informed of the nature of the surgery?	200	90.5	21	9.5
Were you informed of the indication for surgery?	215	97.28	6	2.72
Were you informed of the possible complications of the surgery?	91	41.2	130	58.8
Were you informed of the alternatives of the surgery?	90	40.72	131	59.28
Were you informed of possible complications if surgery is not done?	150	67.87	71	32.13
Were you informed of the expected benefits of surgery?	184	83.25	37	16.75
Were you informed of the type of Anaesthesia?	129	58.37	92	41.63
Were you given an opportunity to have your questions answered?	195	88.23	26	11.77
Did the informed consent influence your decision to undergo surgery?	25	11.31	196	88.69
Were you influenced by anyone to proceed with the surgery?	21	9.5	200	90.5
Are you in favour of the process of informed consent?	216	97.73	5	2.27
Is there any medicolegal significance of the informed consent?	85	38.46	136	61.54
Were you satisfied with the information provided?	213	96.38	8	3.62

The type of anaesthesia for elective surgery was informed to 129 (58.37%) patients, while 92 patients (41.63%) were unaware of it. Two hundred patients (90.5%) answered that they were not influenced by others, such as the surgeons/anaesthesiologists/next of kin or relatives, to proceed with the surgery, and it was their own decision. Two hundred and sixteen (97.73%) patients favoured the informed consent process, and 213 (96.38%) were satisfied with the information provided in the consent form. Regarding if informed consent bears medicolegal significance, only 85 (38.46) patients answered affirmatively, while 136 (61.54%) were unaware of the implications of informed consent.

The content of the informed consent forms was read to know whether complete and appropriate information about the proposed elective procedure or surgery is given to the patients. Results of the same are mentioned in Table 3. The surgical diagnosis was mentioned in only 34% of the consent forms. The type of the procedure, its details, and the risks involved were mentioned in 120 (54.3%) and two (0.9%) of the cases, respectively. The information regarding the type of anaesthesia is mentioned in 54 (24.4%) of the ICFs. Two hundred and fourteen (96.8%) ICFs mentioned the operating surgeon’s name. Of the total 221 ICFs analysed, the signature of the patient and the witnesses was present in 217 (98.2%) and 106 (47.9%) of the IC forms. The name and the signature of the doctor who has administered and discussed the informed consent forms are mentioned in 215 (97.3%) cases.

**Table 3:** Adequacy and completeness of the information in the Informed Consent Form (ICF)

Statements regarding adequacy and completeness of information in the ICF	Yes (%)	No (%)
Is the name of the patient mentioned?	220 (99.5)	01 (0.05)
Is the diagnosis mentioned?	75 (34)	146 (66)
Has the date of the procedure been mentioned?	124 (56.1)	97 (43.9)
Has the procedure been described?	120 (54.3)	108 (45.7)
Have the risks of the procedure been described?	2 (0.9)	219 (99.1)
Has the operating surgeon’s name been mentioned?	214 (96.8)	7 (3.2)
Is information regarding the kind of anaesthesia and specific agents for anaesthesia mentioned?	54 (24.4)	167 (75.6)
Has the patient signed/given a thumb impression on the informed consent form?	217 (98.2)	4 (1.8)
Have the witnesses signed/given a thumb impression on the informed consent form?	106 (47.9)	115 (52.1)
Is the name and the signature of the person who has administered the informed consent mentioned in the informed consent form?	215 (97.3)	6 (2.7)

## Discussion

During this study, 221 patients were interviewed, with a higher percentage of female patients than male patients, with an M:F ratio of approximately 2:3. This is consistent with the fact that more Female surgical patients are in the hospital than male patients. The study participants were between 18 and 80 years old, with the highest percentage being patients between 18-27 years old (26.6%). However, a study by Amir et al. [27] and Tamire and Tesfaw on surgical informed consent had the majority of the patients aged 25-38 years, indicating that most surgical patients are younger [27,28].

The education status of the participating patients varied, with nearly 15.8% being illiterate while 35.3% were educated till the 8th standard (high school). The education status of the patient is essential during the pre-operative counselling and during the IC process, as patients may need help understanding medical terminology used by treating teams, including surgeons and anaesthesiologists. Furthermore, patients with no or little formal education and low literacy are disadvantaged due to their inability to read the informed consent forms [27], affecting comprehension of the information provided during the IC process.

The nature and indication of the proposed surgical treatment were explained to the majority of the patients (92% and 98%, respectively) as part of the informed consent process (ICP), which was similar to another study [27,28]. It is well-known that every invasive procedure involves associated potential risks, and it is the legal and ethical duty of the operating surgeon to make patients aware of both the benefits and risks of the surgery/procedure with equal emphasis. In our study, the potential benefits and risks associated with surgery were informed to 83.25% and 41.2% of the patients.

Tamire and Tesfaw observed that 48.9% of patients were informed of the benefits of surgery, but only 56.1% were informed of the possible complications that may arise following surgery [28]. The importance of sharing specific information about the surgery and its complications well explained empowers patients to make thoughtful decisions after weighing the risks-benefits associated with the proposed surgery [29-35].

In the present study, the alternative treatment options to the consented operative procedure were informed to only 91 (40.72%) patients, while the same was observed in 47.5% of patients by Tamire and Tesfaw [28]. As a part of the informed consent process, alternative modes of treatment, either conservative or surgical, with their associated benefits and risks, should be explained to the patients. The patients shall then freely explore all the available treatment options and, after shared decision-making with the operating surgeon, select the most appropriate treatment option for themselves. The informed consent process is interactive and non-patronising. It involves a dialogue where patients can have their questions answered. This helps assess the patient’s competence to provide the consent and also their understanding of the information provided rather than merely signing the informed consent form.

In our study, about 208 (94.1%) of the patients stated that they understood all the information provided in the ICF, and 213 patients (96.3%) were satisfied with it. Tamire and Tesfaw [28] similarly observed this in 54% and 93.5% of patients. Patient satisfaction with the provided information was seen in 93.5% of patients despite their poor understanding of the informed consent. This was also a strange observation that Amir et al. noted [27].

The preference for the decision to undergo the planned surgery was explored among the surveyed patients, and it was observed that the majority of the patients (88.7%) exercised autonomy in the decision to undergo surgery. Though most of the patients had education up to secondary school, freedom in the decision-making may be possible due to a high level of understanding and satisfaction about the information given during the informed consent process by the care provider teams. Brezis et al., in their study, observed that almost 60% of patients favoured a shared decision-making approach, and nearly 20% preferred an autonomous decision. However, no consistent correlation was observed between the preferred mode of the decision and the patient’s age, education, ethnicity, or setting [36].

The principle of informed consent arises from autonomy or self-determination in the decision-making process. However, patients may have different viewpoints ranging from absolute independence in the decision-making to shared decisions or paternalistic approaches. Clinical ethicists suggest that the informed consent process should be patient-specific and that every course in the decision-making is legitimate [37].

Most patients (97%) favoured the IC process, of which 38.46% believed that informed consent has a medicolegal significance. Similar observations were made in the study by Amir et al. [27]. The comprehensive analysis of the quality of informed consent forms which supports an informed decision revealed critical shortcomings owing to lack of information. For example, it is a requirement that the diagnosis of the diseases for which the elective procedure is planned should be mentioned in the ICF. However, this was not mentioned in 146 (66%) of the cases in the present study. 

The information regarding the kind of anaesthesia and the use of specific agents for anaesthesia were not mentioned in 75.6% of the consent forms. It is agreed that IC forms with less information and inadequate communication of risks are unsuitable for supporting informed decisions. They do not or only partly meet the principles of informed consent for evidence-based health information [38]. The essential legal ingredient of valid informed consent is that a disinterested party should witness it [39]. Some authors suggest the following duties of a witness to enhance the protection of the research subjects: The witness should verify (a) the accuracy of information provided to the subject, (b) the subject’s clear understanding of the information disclosed, and (c) the subject’s voluntary agreement to take part in the investigation [40]. But in this study, only 47.9% of the ICFs were signed by the witnesses, which may pose legal and ethical challenges.

Study limitations

This study has some limitations in that most patients were made aware of the present study being conducted, which may have influenced their responses to our survey questions (Hawthorne effect). It may be possible that the patients who did not have a satisfactory outcome after surgery may have responded to our questionnaire unfavourably or refused to participate, thus limiting the patient pool which responded to our questionnaire to those patients who had a satisfactory surgical outcome, which may have influenced our results. Further, we had no a priori sample size calculation done and we selected consecutive patients who consented to elective surgery and were usually admitted a day before or had already recently undergone elective surgery. Despite these limitations, we have sincerely attempted to understand the adequacy of informed consent in elective surgical procedures at a tertiary teaching hospital and research centre and have found some important inadequacies and concerns.

## Conclusions

The present study analysed the informed consent process using patients’ responses to the question statements and direct observation of the information presented in the informed consent documents. It was observed that most patients were explained the nature and indication of the planned surgical operation/procedure. The study revealed that for better decision-making capacity of the patients, it is vital to share the potential benefits and risks associated with surgery. The present study emphasises informed consent’s significance before a planned surgical procedure. However, the lack of information in the informed consent forms critically affects the quality and adequacy of the IC and is not suitable for supporting a well-informed and autonomous decision-making process, thus posing ethical and legal challenges for truly informed consent. Thus, further audits and policy changes are warranted to improve the IC process.

## Appendices

Data capture sheet
